# Early versus late atrial fibrillation recurrence after pulsed field ablation: insights from the admIRE trial

**DOI:** 10.1093/europace/euaf007

**Published:** 2025-01-16

**Authors:** Luigi Di Biase, Vivek Y Reddy, Marwan Bahu, David Newton, Christopher F Liu, William H Sauer, Sandeep Goyal, Vivek Iyer, Devi Nair, Jose Osorio, Moussa Mansour, Hugh Calkins, Oussama Wazni, Andrea Natale

**Affiliations:** Cardiac Arrhythmia Center, Division of Cardiology at the Montefiore Medical Center, Albert Einstein College of Medicine, 111 E 210 St., New York, NY 10467, USA; Helmsley Electrophysiology Center, Mount Sinai Fuster Heart Hospital, New York, NY, USA; Phoenix Cardiovascular Research Group, Phoenix, AZ, USA; Clinical Cardiac Electrophysiology Department, Memorial Health, Savannah, GA, USA; Cardiology Department, Weill Cornell Medicine—New York Presbyterian Hospital, New York, NY, USA; Cardiac Arrhythmia Service, Brigham and Women’s Hospital and Harvard Medical School, Boston, MA, USA; Piedmont Heart of Buckhead Electrophysiology, Piedmont Heart Institute, Atlanta, GA, USA; MarinHealth Cardiovascular Medicine, Marin Health Medical Center, Larkspur, CA, USA; Heart & Vascular Department, St. Bernards Medical Center & Arrhythmia Research Group, Jonesboro, AR, USA; HCA Florida Miami Electrophysiology - Cardiovascular Group, HCA Florida Miami, Miami, FL, USA; Atrial Fibrillation Program, Massachusetts General Hospital, Boston, MA, USA; Electrophysiology Laboratory and Arrhythmia Service, Johns Hopkins Medical Institutions, Baltimore, MD, USA; Cardiac Electrophysiology and Pacing, Cleveland Clinic Foundation, Cleveland, OH, USA; Texas Cardiac Arrhythmia Institute, St. David’s Medical Center, Austin, TX, USA; Department of Biomedicine and Prevention, Division of Cardiology, University of Tor Vergata, Rome, Italy

**Keywords:** Early recurrence, Late recurrence, Atrial fibrillation, Ablation, Pulsed field ablation, Varipulse

## Abstract

**Aims:**

Studies have shown correlations between early recurrence (ER) and late recurrence (LR) of atrial arrhythmia after ablation with thermal technologies. This admIRE trial (NCT05293639) subanalysis aims to analyse ER vs. LR in patients with paroxysmal atrial fibrillation (PAF) undergoing pulsed field ablation (PFA).

**Methods and results:**

Patients with symptomatic paroxysmal atrial fibrillation and ≥1 transtelephonic monitoring transmission during the blanking period were included (*n* = 169). ER was defined as documented recurrence in the blanking period (days 1–90), and LR as recurrence in the evaluation period (days 91–365). Freedom from 12-month recurrence was estimated using Kaplan–Meier method. A Cox proportional-hazards regression model, with ER as the primary factor, and adjusted for age, sex, and body mass index, was used to estimate hazard ratios (HRs) and 95% CI. ER was observed in 20.1% (31/169) of patients (66.1 ± 7.1 years, 35.5% female, 46.6 ± 48.4-month PAF history). Time to first documented ER was 49 (37–61) days. Occurrence of LR was 16.7% (23/138) in patients without ER, 71.0% (22/31) in those with ER, and 87.0% (20/23) in patients whose ER onset occurred within the first 2 months. Twelve-month freedom from documented recurrence was significantly lower in patients with ER at 29.0% (95% CI, 13.1–45.0%) vs. 82.5% (95% CI, 75.9–89.1%) in those without ER (adjusted HR, 7.9; 95% CI, 4.1–15.1; *P* < 0.001).

**Conclusion:**

This admIRE subanalysis demonstrated that PAF patients who experience ER after PFA are at a substantially higher risk for LR. The optimal duration of the blanking period post-PFA needs further assessments.

**ClinicalTrials.gov Identifier:**

NCT05293639

## Graphical Abstract

**Graphical Abstract:**
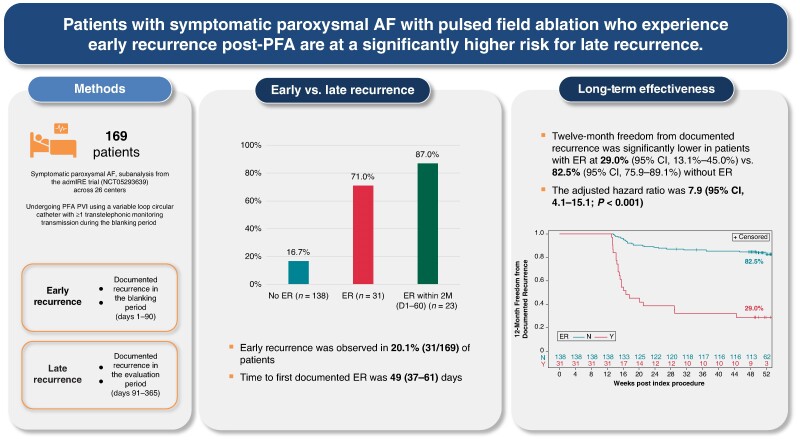

## Introduction

Early recurrence (ER) post–catheter ablation may occur and impact the overall success and prognosis of the procedure. Understanding the implications of ER and its relationship to late recurrence (LR) is crucial for improving patient outcomes and guiding clinical decision-making.

Studies have shown correlations between ER and LR after catheter ablation with thermal technologies.^1–6^ Recurrence during the first 3 months post-ablation has been shown to be a predictor of long-term recurrence using radiofrequency (RF) technologies in patients with atrial fibrillation (AF).^4,5^ Similar results were observed in cryoballoon studies,^2,3^ leading to the early suggestions of shortening the blanking period from the previously defined 3 months. The CIRCA-DOSE trial demonstrated that ER taking place three weeks post-ablation was a predictor for LR.^1^ There is scarce evidence on the correlation between ER and LR in patients being treated with pulsed field ablation (PFA). Thus far, only the PULSED AF trial and an additional case series of patients with AF undergoing PFA showed a positive correlation between ER and LR in subanalyses.^7,8^

The present subanalysis therefore aims to analyse early vs. late atrial arrhythmia recurrence in patients with paroxysmal atrial fibrillation (PAF) undergoing PFA using a variable loop PFA catheter.

## Methods

### Study design and ablation procedure

The admIRE trial (NCT05293639) was a multicenter, prospective, nonrandomized, single-arm interventional study to assess the safety and effectiveness of a variable loop PFA catheter (Varipulse Catheter, Biosense Webster Inc., part of Johnson & Johnson MedTech, Irvine, CA, USA) with an integrated electroanatomical mapping system (CARTO Software, Biosense Webster Inc., part of Johnson & Johnson MedTech, Irvine, CA, USA). Full details of the admIRE trial study design and ablation procedure have been previously reported.^9^ The study catheter was utilized for pulmonary vein isolation. If additional left atrial arrhythmias were identified during the index procedure, ablation outside of the pulmonary veins was allowed using the study catheter for segmental application delivery (≥6 electrodes or circumferential applications with 10 electrodes). In some cases with typical right atrial flutter, cavotricuspid isthmus (CTI) linear ablation using a commercially approved radiofrequency ablation catheter was authorized. The institutional ethics review boards at each study center approved the study, and all enrolled patients provided informed consent prior to moving forward with study procedures.

### Patient follow-up and monitoring

Monitoring for arrhythmia recurrence was previously described in detail.^9^ Transtelephonic monitoring (TTM; KardiaMobile 6L, AliveCor, Mountain View, CA, USA) was performed weekly from months 1 to 5, monthly from months 6 to 12, and for symptomatic events. TTM transmissions for months 1 and 2 were optional per the study protocol. A 12-lead electrocardiogram was completed at months 3, 6, and 12, and 24-hour Holter monitoring was also performed at months 6 and 12. All recordings were reviewed by an independent core laboratory.

### Definitions and study outcomes

ER was defined as documented recurrence in the blanking period (days 1–90), while LR was defined as documented recurrence in the evaluation period (days 91–365). The primary endpoint for this subanalysis was LR in patients who exhibited ER in comparison to those who did not.

### Statistical analysis

Baseline and procedural characteristics were presented descriptively as median and interquartile range (IQR) or count and percentage, and compared by means of Wilcoxon rank-sum test or Fisher’s exact test, as appropriate. Freedom from recurrence at 12 months was estimated using the Kaplan–Meier method. A Cox proportional-hazards regression model, with ER as the primary factor and LR as the dependent variable, adjusted for age, sex, body mass index, AF duration, cardiomyopathy, and LA diameter was used to estimate hazard ratio (HR) and 95% CI. Statistical analyses were performed using SAS 9.4 or SAS Studio 3.8 (SAS Institute, Inc, Cary, NC, USA).

## Results

### Patient characteristics with or without early recurrence

A total of 169 patients from 26 sites with symptomatic paroxysmal atrial fibrillation and ≥1 TTM transmission during the blanking period was included in this analysis. The median age was 65 (IQR 58–68) years, with a median time since diagnosis of 24 (IQR 12–70) months, and 35.5% were female. Early recurrence was observed in 18.3% (31/169) of patients whose characteristics were comparable to those of patients without ER (*n* = 138), with the exception of age [68.0 (62.0–72.0) vs. 64.0 (57.0–68.0) years] and CHA_2_DS_2_–VASc [2.0 (1.0–3.0) vs. 1.0 (1.0–3.0)], *Table 1*. The median (IQR) time to first documented ER was 49 (37–61) days.

**Table 1:** Baseline characteristics

Characteristics^a^	ER^b^ (*n* = 31)	No ER (*n* = 138)	*P*-value
Age, years	68.0 (62.0–72.0)	64.0 (57.0–68.0)	0.001
Female, %	11 (35.5)	49 (35.5)	>0.999
Body mass index	28.0 (24.9–33.7)	27.8 (24.3–30.8)	0.343
LVEF, %	61.0 (57.0–65.0)	60.0 (55.0–65.0)	0.375
Left atrial diameter, mm	39.0 (36.0–44.0)	38.0 (35.0–43.0)	0.217
CHA_2_DS_2_–VASc	2.0 (1.0–3.0)	1.0 (1.0–3.0)	0.027
PAF history, months	24.0 (12.0–72.0)	24.6 (12.0–67.0)	0.940
Myocardial infarction	3 (9.7)	3 (2.2)	0.076
Hypertension	21 (67.7)	72 (52.2)	0.161
Type 2 diabetes	8 (25.8)	13 (9.4)	0.029
Coronary disease	9 (29.0)	28 (20.3)	0.337
Obstructive sleep apnoea	9 (29.0)	39 (28.3)	>0.999
Thromboembolic events	3 (9.7)	4 (2.9)	0.116
Congestive heart failure	1 (3.2)	5 (3.6)	>0.999
NYHA class I	0 (0.0)	1 (0.7)	>0.999
NYHA class II	1 (3.2)	4 (2.9)	
Number of failed AADs	1.0 (1.0–1.0)	1.0 (1.0–1.0)	0.664
Class I/III AAD	1.0 (1.0–1.0)	1.0 (1.0–1.0)	0.368

ER, early recurrence; PAF, paroxysmal atrial fibrillation.

^a^Data are presented as median (IQR) or *n* (%).

^b^ER: 3-month (days 0–90) recurrence.

### Procedural characteristics

The procedure time, mapping time, and fluoroscopy times between patients with vs. without ER were comparable: 96.0 (86.0–120.0) vs. 98.5 (72.0–121.0), 6.0 (4.0–11.0) vs. 7.0 (5.0–10.0), and 6.1 (1.1–14.1) vs. 6.6 (0.0–13.4), respectively (*Table 2*). Intracardiac echocardiography was used in about 94% of cases in each cohort. The number of valid PFA applications per patient were also similar with 65.0 (58.0–73.0) in the ER group and 70.0 (60.0–85.0) in the non-ER group.

**Table 2:** Procedural characteristics

Characteristics^a^	ER (*n* = 31)	No ER (*n* = 138)	*P*-value
Procedure time, min	96.0 (86.0–120.0)	98.5 (72.0–121.0)	0.419
Procedure time for PVI, min	93.0 (86.0–112.0)	82.0 (64.0–114.0)	0.201
Total mapping time, min	6.0 (4.0–11.0)	7.0 (5.0–10.0)	0.673
Transpired PFA time, min	31.9 (27.5–43.9)	31.9 (25.7–41.2)	0.468
Fluoroscopy time, min	6.1 (1.1–14.1)	6.6 (0.0–13.4)	0.865
Cases performed without fluoroscopy	6 (19.4)	35 (25.4)	0.644
Cases in which ICE was used	29 (93.6)	129 (93.5)	<0.999
Cases with only the study catheter for mapping	1 (3.2)	13 (9.4)	0.470
Number of valid PFA applications per patient	65.0 (58.0–73.0)	70.0 (60.0–85.0)	0.182
Received additional posterior wall segmental ablation	1 (3.2)	14 (10.1)	0.310
Received additional CTI ablation	5 (16.1)	23 (16.7)	>0.999

ER, early recurrence; ICE, intracardiac echocardiography; PFA, pulsed field ablation; PVI, pulmonary vein isolation.

^a^Data are presented as median (IQR) or *n* (%).

A total of 1 (3.2%) of patients in the ER group and 14 (10.1%) in the non-ER group received additional posterior wall segmental ablation. In addition, 5 (16.1%) and 23 (16.7%) received additional cavotricuspid isthmus ablation (*Table 2*).

### Early versus late recurrence correlation

**Figure 1:**
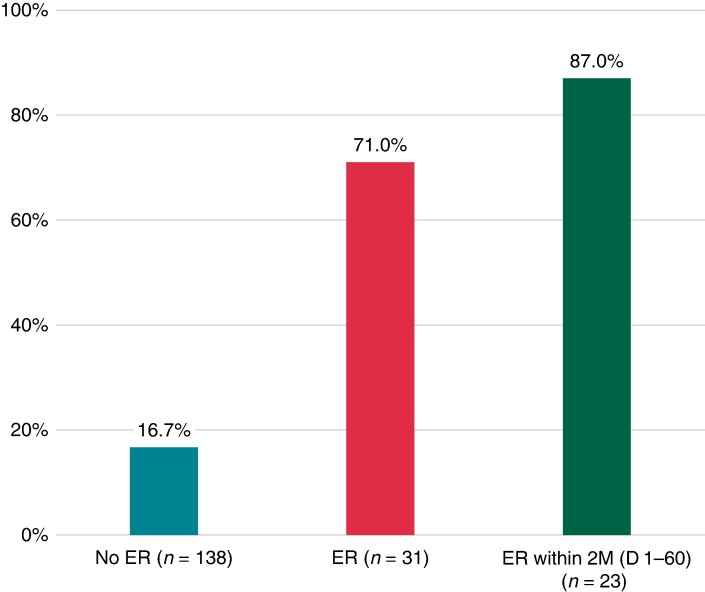
Proportion of patients with LR (post–blanking period). Proportions of patients with LR among patients with no ER, ER, and ER within 2 months. ER: 3-month (days 0–90) recurrence. LR: 3–12 month (days 91–365) recurrence. ER, early recurrence; LR, late recurrence.

**Figure 2:**
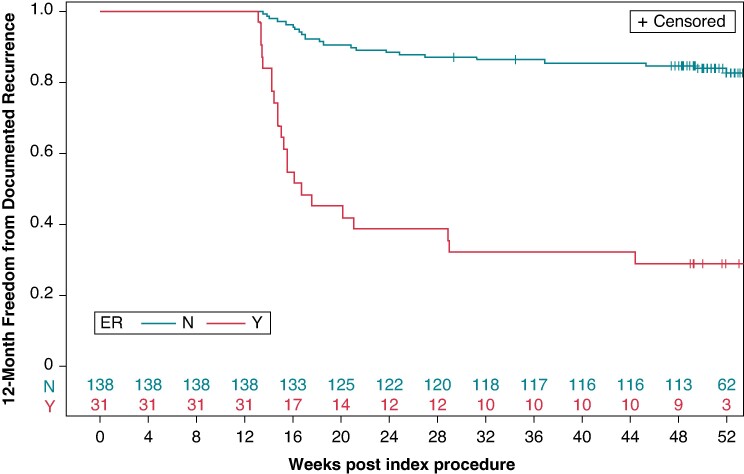
Freedom from documented recurrence in the evaluation period (days 91–365). Freedom from recurrence at 12 months estimated using the Kaplan–Meier method for patients with and without ER. ER: 3-month (days 0–90) recurrence. ER, early recurrence; N, no; Y, yes.

The occurrence of LR was 16.7% (23/138) in patients without ER. Late recurrence occurrence was 71.0% (22/31) in those with ER and 87.0% (20/23) in patients whose ER onset occurred within the first 2 months (*Figure 1*). The 12-month freedom from documented atrial tachyarrhythmia recurrence was found to be significantly lower in patients with ER at 29.0% (95% CI, 13.1–45.0%), compared with 82.5% (95% CI, 75.9–89.1%) in those without ER (adjusted HR, 7.9; 95% CI, 4.1–15.1; *P* < 0.001, *Figure 2*).

Further analysis to understand the negative predictive values (NPV) and positive predictive values (PPV) were performed (*Table 3*). The PPV at 2-month ER was 87.0 (73.2–100.0) compared to 3-month ER with a PPV of 71.0 (55.0–87.0). In addition, the NPV for 2-month ER was 82.5 (76.3–88.7) vs. 3-month ER at 83.0 (76.6–89.3).

**Table 3:** PPV and NPV

Statistic	Estimates^a^	
	2M ER	3M ER
Sensitivity	44.4 (29.9–59.0)	48.9 (34.3–63.5)
Specificity	97.5 (94.8–100.0)	92.6 (87.9–97.2)
PPV	87.0 (73.2–100.0)	71.0 (55.0–87.0)
NPV	82.5 (76.3–88.7)	83.0 (76.6–89.3)

CI, confidence interval; ER, early recurrence; M, month; NPV, negative predictive value; PPV, positive predictive value.

^a^Data are presented as percent estimate (95% CI).

### AAD utilization

About 5.3% (9/169) of patients took a higher dose or new Class I/III antiarrhythmic drugs (AADs) during the blanking period (two on higher than the previously failed dose and seven on new AAD). A total of five ER episodes across five patients were treated with AADs (four with a new AAD and one with a higher dose of a currently taken AAD). Five patients were taking oral anticoagulants starting on Day 1, unrelated to ER. Over the course of the 12-month follow-up period, 15.7% patients were on Class I/III AADs during months 1–3, 8.7% during months 3–6, and 6.5% during months 6–12, regardless of ER.

### Safety

Procedural complication rates, defined as adverse events on the date of the index procedures, between with vs. without ER were 16.1% (5/31) and 6.5% (9/138), respectively. All five of the procedural complication events in the ER group were procedure-related, while five out of the nine without ER were procedure-related. The primary adverse event rates between the two groups were 6.5% (2/31) in the ER group and 1.5% (2/138) in the LR group.

## Discussion

The findings of this admIRE trial subanalysis evaluated the incidence and timing of ER in patients with PAF following PVI with PFA and its effect on long-term success, as indicated by freedom from LR over the course of 12 months. We have demonstrated the significance of ER as a predictor of reduced long-term effectiveness.

Larger studies analysing the correlation between ER and LR in patients with paroxysmal atrial fibrillation (PAF) and persistent AF are needed to fully understand the possibility to reduce, and potentially eliminate, the blanking period post-PFA. Awareness of this relationship holds promise in improving patient quality of care.

In this subanalysis of the admIRE study, which included 31 patients with ER, the occurrence of LR was substantially lower in patients without ER (16.7%) than in those with ER (71%) or ER onset within the first 2 months (87%). Twelve-month freedom from documented atrial tachyarrhythmia recurrence was significantly higher in patients without ER (84.0%) than in those with ER (29.0%; *P* < 0.001). Class I/III AAD use was relatively low (<6%) during the blanking period. In an analysis to understand the NPV and PPV, there was a high level of comparability in the NPV regardless of the duration of blanking period (2-month ER, 82.5 [76.3–88.7]; 3-month ER, 83.0 [76.6–89.3]).

Similar results to those shown in the current study were observed in a previous secondary analysis of the PULSED AF study, which evaluated the safety and efficacy of a PFA system (PulseSelect Pulsed Field Ablation System; Medtronic, Minneapolis, MN, USA) for treatment of paroxysmal and persistent AF.^7^ Their secondary analysis, which used similar definitions for ER (recurrence within 90 days post-ablation) and LR (recurrence between 90 days and 12 months post-ablation), included 154 patients with PAF and 140 patients with persistent AF, 27% and 32% of whom, respectively, had ER.^7^ Overall, the occurrence of LR was lower in patients with no ER (24%) than in those with ER (67%), and this difference was even more pronounced in the subgroup with PAF (no ER, 20%; ER, 71%).^7^ The incidence of LR was higher in patients with their last ER episode during 61–90 days post-ablation (83% overall; 79% for PAF) than in those with their last episode within 1–30 days (47% overall; 67% for PAF) or 31–60 days (42% overall; 56% for PAF).^7^

In addition, Mohanty *et al.*^8^ analysed 337 patients undergoing PFA for AF. Early recurrences were recorded in 15.7% patients. At 1-year follow-up, all patients with recurrence in the second and third months experienced late recurrence. Therefore, the authors concluded that early recurrence in the second or third month after the PFA procedure was associated with a high risk of late recurrence. Thus, the authors suggested that the blanking period could be redefined as little as 1 month after PFA.

Similar analyses have been performed in patients undergoing RF or cryoballoon ablation for AF.^1,3,4^ In an analysis including 207 consecutive patients with persistent AF, in which ER was defined as recurrence within the first 30 days post-ablation, ER occurred in 69.1% of patients.^4^ LR, which was defined as recurrence >30 days post-ablation, occurred significantly more frequently in patients with ER (92.3%) than in those without ER (43.8%; *P* < 0.001).^4^ Also, in a registry study of 2,636 patients with paroxysmal or persistent AF undergoing cryoballoon ablation, in which ER was defined as recurrence within 90 days post-ablation and LR was recurrence >90 days post-ablation, the 1-year freedom from LR was significantly lower in patients with ER (42.6%) than in those without ER (85.5%).^3^ Among patients with ER, the 1-year freedom from LR was 44.1% for patients with ER within 30 days post-ablation, 32.4% for ER between 30 and 60 days post-ablation, and 39.0% for ER between 60 and 90 days post-ablation (*P* = 0.051).^3^ Similar results were shown in the CIRCA-DOSE trial comparing RF ablation with cryoballoon ablation in 346 patients with PAF, in which 61% of patients had ER (defined as recurrence within 90 days post-ablation).^1^ A significantly higher proportion of patients with ER than without ER experienced LR (*P* < 0.001), and later ER episodes (during the third month after blanking) were associated with a higher likelihood of LR than ER episodes in the first or second month.^1^

The analyses of timing of ER and occurrence of LR in the PULSED AF secondary analysis in patients undergoing PFA^7^ and similar analyses in patients undergoing RF or cryoballoon ablation^1,3,4^ indicate that ER within the third month post-ablation may be considered LR and have led to the recommendation to shorten the blanking period post-ablation. A recent subanalysis using RF in patients with persistent atrial fibrillation also discovered substantially increased long-term arrhythmia recurrence in patients with recurrence during the blanking period compared to those without.^10^ The corresponding editorial comments on growing evidence to shorten the blanking period to two months.^11^ Since then, the 2024 European Heart Rhythm Association (EHRA)/Heart Rhythm Society (HRS)/Asia Pacific Heart Rhythm Society (APHRS)/Latin American Heart Rhythm Society (LAHRS/SOLAECE) guidelines recommend a reduced 2-month blanking period^12^ compared with the 3-month blanking period recommended in the 2017 HRS/EHRA/European Cardiac Arrhythmia Society/APHRS/SOLAECE guidelines.^13^ The 25 years of progress in atrial fibrillation review emphasizes the potential for further improvements in AF mapping and technologies to propel these clinical outcomes, such as reduction in AF recurrence, for patients treated with PFA.^14^ Whether this new ablation modality can be considered in the same respect as previous technologies, such as radiofrequency and cryoballoon, is still under further analysis. So far, a propensity score matching study found similar long-term efficacy across the three modalities.^15^ Additional therapies, including high power, short duration, have also been considered and have shown comparable recurrence rates.^16^ Whether these findings translate into reducing the blanking period is up to further question. In the current study, ER within the 2-month blanking period predicted LR more effectively compared to ER within the protocol-defined 3 months. This higher rate suggests that ER even early on can predict LR, calling into question a 2-month or perhaps a nonexistent blanking period. Nevertheless, results of the current analysis support the association between ER and LR observed in previous studies. This subanalysis may shape future studies to investigate the need for a blanking period using different modalities.

Additional studies on AF ablation treatment have investigated predictors of ER vs. LR, such as C-reactive protein or early pulmonary vein reconnection.^17,18^ While pulmonary vein reconnection found about 30 min post-ablation was associated with LR, the C-reactive protein had no significant relationship to neither ER nor LR. Further investigations into predictors for ER vs. LR are warranted.

### Limitations

The admIRE trial did not require TTM during the protocol-defined blanking period (months 1–3). Patients with a transmitted recording were likely to be symptomatic and/or more compliant to the study protocol. The sample size therefore would have represented a larger proportion of the overall enrolled patient population if remote monitoring was required during the blanking period. The overall low number of ER events prevented us from performing a time-sensitive analysis stratifying the impact of ER during the first month vs. recurrences during the second month post–blanking period.

Furthermore, not all patients received their TTM devices within the first month after the index ablation. This was due to issues surrounding vendor shipping.

## Conclusion

This admIRE subanalysis demonstrated that symptomatic PAF patients who experience ER after PFA are at a substantially higher risk for LR. These data underscore the importance of early post-ablation monitoring and may inform future strategies to mitigate ER and improve long-term clinical outcomes. The optimal duration of the blanking period post-PFA requires further assessment.

## Data Availability

Johnson & Johnson MedTech has an agreement with the Yale Open Data Access (YODA) Project to serve as the independent review panel for the evaluation of requests for clinical study reports and patient-level data from investigators and physicians for scientific research that will advance medical knowledge and public health. Requests for access to the study data can be submitted through the YODA Project site at http://yoda.yale.edu.
